# Be there on time: Spatial‐temporal regularities guide young children’s attention in dynamic environments

**DOI:** 10.1111/cdev.13770

**Published:** 2022-04-06

**Authors:** Nir Shalev, Sage Boettcher, Hannah Wilkinson, Gaia Scerif, Anna C. Nobre

**Affiliations:** ^1^ 6396 Department of Experimental Psychology University of Oxford Oxford UK; ^2^ 6396 Oxford Centre for Human Brain Activity, Wellcome Centre for Integrative Neuroimaging, Department of Psychiatry University of Oxford Oxford UK

**Keywords:** attention, cognitive development, visual search

## Abstract

Children's ability to benefit from spatiotemporal regularities to detect goal‐relevant targets was tested in a dynamic, extended context. Young adults and children (from a low‐deprivation area school in the United Kingdom; *N* = 80; 5–6 years; 39 female; ethics approval did not permit individual‐level race/ethnicity surveying) completed a dynamic visual‐search task. Targets and distractors faded in and out of a display over seconds. Half of the targets appeared at predictable times and locations. Search performance in children was poorer overall. Nevertheless, they benefitted equivalently from spatiotemporal regularities, detecting more predictable than unpredictable targets. Children's benefits from predictions correlated positively with their attention. The study brings ecological validity to the study of attentional guidance in children, revealing striking behavioral benefits of dynamic experience‐based predictions.

**Abbreviations**
GLMMs: generalized linear mixed‐effects models
LMM: linear mixed‐effect model
RT: reaction times
SWAN: Strengths and Weaknesses of ADHD‐symptoms and Normal‐behaviors

## INTRODUCTION

Many tasks in real‐life situations require us to manage multiple dynamic sources of information successfully. However, even for neurotypical adults, it is impossible to process all concurrent stimuli in complex and dynamically changing environments in their entirety. Instead, to behave adaptively, it is necessary to prioritize and select task‐relevant information according to our goals and to reject irrelevant, distracting information. For example, crossing a busy road requires paying attention to a changing and hazardous environment while selectively prioritizing critical signals at different locations and timings (traffic lights, car signals, etc.) and ignoring equally salient signals (advertisement boards, flying birds, etc.).

Avoiding hazards can be particularly challenging for children. Thus, we would be alarmed if a 5‐year‐old was to cross a busy street on her own, given children's proposed reduced capacity to ignore distraction and to focus on relevant information efficiently (Amso & Scerif, 2015; Colombo, 2001; Johnson, 2001; Scerif, 2010). Salient events tend to capture children's attention (e.g., a noisy motorbike) and divert them from goal‐relevant streams of information (e.g., the traffic on the road) (Gaspelin et al., 2015). The presence of multiple competing perceptual events is likely to exacerbate the problem (Kim & Kastner, 2019).

When studying selection, competition, and distraction; visual‐search tasks are a popular choice (Wolfe, 2020). In a typical visual‐search task, observers search for a target appearing among a static display of distractors. In such tasks, several sources of information have been shown to guide attention (Wolfe & Horowitz, 2017). Target information will guide attention to objects that share features with the target through goal‐directed, top‐down signals. Intrinsically salient events also attract attention through bottom‐up mechanisms (Theeuwes, 2018; Theeuwes & Burger, 1998). Finally, memories of different types and time scales also contribute to guidance (see Nobre & Stokes, 2019). Notably, selection history during search has been proposed to guide attention to attributes or areas which experience indicates are predictive of the target (Awh et al., 2012; Chun & Jiang, 1998; Geng & Behrmann, 2002, 2005).

It is widely recognized among developmental researchers that children perform poorly in visual‐search tasks compared to adults (Donnelly et al., 2007; Trick & Enns, 1998). Proficiency in searching for targets among distractors with different combinations of the same features develops gradually into adolescence, with a marked improvement at around 6–7 years (Hommel et al., 2004; Lobaugh et al., 1998; Merrill & Lookadoo, 2004; Ólafsdóttir et al., 2016; Whitebread & Neilson, 2000). These age‐related differences are often attributed to the interaction between immature top‐down attention‐control mechanisms and developing sensory processes (Donnelly et al., 2007; Kim & Kastner, 2019). Studies of visual search in children, therefore, suggest a core limitation of the developing mind in controlling goal‐directed attention (Merrill & Lookadoo, 2004; Trick & Enns, 1998).

The ability of selection history to contribute to children's search performance is less well established. In some types of search tasks, children can learn about repeating regularities that aid performance. For example, in Contextual Cueing tasks, observers are better at finding targets that appear among spatial distractors when the same array configuration repeats multiple times (Chun & Jiang, 1998). The repeated exposure to the same arrangement of stimuli serves as a memory cue that guides individuals, including school‐aged children (age 8–12 years old), toward the target location more rapidly (Darby et al., 2014). However, the findings in relation to children's sensitivity to contextual cueing are often conflicting. Some studies indicate memory‐guided performance among school‐aged children of various age groups (ages: 5–9, 6–9, and 9–13 years old) to be comparable to that of adults (Dixon et al., 2010; Yang & Song, 2021), while other studies find reduced learning capacity in school‐aged children (ages: 10 and 6–13 years old) (Couperus et al., 2011; Vaidya et al., 2007). The discrepancy between the findings in the developmental literature has been attributed to various task factors such as the use of different types of distractors (Couperus et al., 2011) or the length of the task (Darby et al., 2014), but does not seem to be due to the particular age ranges chosen across the studies. Altogether, the contextual learning literature indicates that, while children are capable of learning about spatial regularities, this capacity may be confined to specific contexts.

Interestingly, outside of visual‐search tasks, children have been proposed to display a relative strength in learning new information, compared to adults. This ability has been proposed to result from an inherent trade‐off between top‐down cognitive control versus exploratory cognitive flexibility (Gopnik et al., 2017). According to this view, gaining life experience alters the way we interact with the environment. Improvements in cognitive control into adulthood are coupled with a tendency to exploit prior knowledge to maximize utility in a given context and to be less exploratory. In contrast, during childhood, exploratory behavior may be more adaptive, serving to amplify learning about novel characteristics of the environment and helping to shape subsequent knowledge structures that come to guide adaptive behavior in the future. Consequently, children outperform adults in tasks requiring the extraction of unusual causal patterns, for example, in learning how to operate a machine based on counterintuitive rules (Lucas et al., 2014) and are also more likely to encode task‐irrelevant or distractor‐related information (Plebanek & Sloutsky, 2017; Sloutsky & Fisher, 2004).

Ultimately, in order to understand how the different qualities of children's cognition guide adaptive behavior, it is necessary to upgrade visual‐search tasks to capture the ecological aspects of real‐life situations. In everyday extended dynamic contexts, the anticipation of events based on previous experience plays a fundamental role in guiding attention proactively to the right place at the right time. In a series of experiments, we recently demonstrated that when performing a visual‐search task that extends over time, young adults can learn spatial and temporal regularities about the appearance of task‐relevant items and then use these predictable patterns to improve performance (Boettcher et al., 2021; Shalev, Boettcher, et al., 2019; Shalev et al., 2020, 2021). Our experimental framework differs from typical studies using informative cues to study event anticipation. Using a dynamic visual‐search task, we embed spatial and temporal predictions within multiple dynamic, competing, and temporally extended events. Knowledge about these spatiotemporal regularities in our task is measured through the benefits that the predictions confer to performance. Participants are not informed about the embedded spatiotemporal regularities or asked about them explicitly as part of the task. In line with proposed nomenclature within timing studies, we refer to our task as measuring implicit spatiotemporal memories guiding behavior (Coull & Nobre, 2008; for a discussion on explicit and implicit use of timing over developmental time, see also Droit‐Volet & Coull, 2016).

Interestingly, in contrast to visual‐search studies, in which children (and even adolescents) often perform poorly when compared with adults (e.g., Donnelly et al., 2007; Trick & Enns, 1998), cueing tasks reveal a different trajectory. Spatial orienting based on simple and salient attention cues is already detectable during infancy (by 4 months) (e.g., Johnson et al., 1991). Although orienting skills continue to develop during infancy, there are limited differences in shifting of attention following exogenous spatial cues (Brodeur & Enns, 1997) and temporal cues (e.g., Droit‐Volet & Coull, 2016) in school‐aged children compared to adults. Indeed, as in spatial attention (Johnson et al., 1991), the ability to anticipate a stimulus based on implicit temporal regularities has been noted as early as infancy, as babies react to the omission of a regular, predictable stimulus (e.g., Brackbill & Fitzgerald, 1972; Colombo & Richman, 2002; Mento & Valenza, 2016). In older age groups, 4‐ and 5‐year‐old children can extract task‐embedded temporal properties to anticipate upcoming events (e.g., Droit‐Volet & Coull, 2016; Johnson et al., 2015; Mento & Granziol, 2020; Vallesi & Shallice, 2007). These studies indicate that young children can guide attention in time. However, the tasks used so far are restricted to testing responses to single targets occurring alone within temporally confined trials, and do not yet address the ability of benefitting from temporal predictions to select targets among competing stimuli in dynamic and temporally extended contexts.

Studies of statistical learning suggest that children are able to encode predictable structures in extended contexts beyond individual cue‐interval associations (see Saffran & Kirkham, 2018; Thiessen, 2017). For example, studies of visual statistical learning have shown children's ability to learn spatial (e.g., Bertels et al., 2017; Tummeltshammer et al., 2017) and sequential (e.g., Fiser & Aslin, 2002; Kirkham et al., 2002; Wu et al., 2011) relations between successive items in a stream. However, statistical learning tasks have not directly tested the ability to learn about the timing of event onsets within extended contexts, which is distinct from their sequential order. They have also not yet incorporated unpredictable distracting stimulation, which is often a feature of natural situations.

In unfolding real‐life tasks, neither spatial nor temporal structures are held constant (as in laboratory search or cueing tasks), and prior experience of environmental regularities is likely contribute to guidance. Whereas previous studies on spatial cueing, control of temporal attention, and statistical learning provide a promising foundation, it remains unclear whether children can extract and utilize concurrent spatial and temporal predictions about the occurrence of relevant events occurring within dynamic contexts and among competing task‐irrelevant distractors. Traditional views, rooted in developmental studies of visual search, emphasize the reduced capacity for goal‐directed attentional guidance early in development. Here, we wished to explore whether children's propensity to learn environmental structures would extend to their ability to learn and utilize dynamic spatiotemporal structures within extended visual search contexts through selection history to predict the occurrence of targets and thereby to support goal‐directed attention.

In the current study, we build on the foundations of static visual search, cued temporal orienting, and statistical‐learning tasks to develop an experimental framework for investigating whether children can benefit from spatiotemporal predictions to guide attention in complex and dynamically changing contexts. This is akin to natural environments changing in space and time—like a busy street. We aim to learn whether spatiotemporal regularities can benefit young observers performing a dynamic visual search. We adapted the dynamic visual search (DVS) task we recently developed to investigate whether children and adults use task‐embedded spatial and temporal regularities to guide selective attention within extended contexts. In our task, target and distractor stimuli fade in and out of a noisy background display over several seconds, with half of the targets appearing predictably at the same onset time and quadrant. In principle, learning of the spatiotemporal regularities can guide anticipatory attention to the location and timing of predictable targets. The experimental approach was developed and validated in a series of experiments testing more than a hundred young adults (Boettcher et al., 2021; Shalev, Boettcher, et al., 2019).

Children and young‐adult participants performed the same dynamic visual search on a digital tablet. To make the task more engaging, pictures of an airplane, dragonfly, and birds acted as targets and distractors. Participants had to detect a pre‐designated target stimulus (e.g., an airplane) among competing distracting stimuli (e.g., dragonflies and birds). On every trial, they indicated where they detected the pre‐designated target by touching its location. The central within‐participant experimental manipulation concerned the spatiotemporal predictability of a subset of target stimuli: Half of the targets consistently appeared at predictable quadrants and onsets, whereas the other half were unpredictable. The experimental task allowed us to compare overall performance as well as the ability to benefit from spatiotemporal predictions in the different age groups (children vs. adults).

We hypothesized that, while children may find fewer targets overall compared to adults, they may nevertheless learn temporal and spatial regularities to guide attention to targets. In addition to looking at age‐related differences between young children (5–6 years old) and adults, we also tested whether the capacity to form spatiotemporal predictions was associated with children's age or with behavioral markers of typical attention development. While informed by prior theory and data obtained with young adults, our analysis was exploratory in nature.

## METHODS

All experimental procedures and protocols were reviewed and approved by the central university research ethics committee of the University of Oxford.

### Participants

#### Adults

The adult sample consisted of 25 undergraduate students (20 female) with mean age 20.08 years (*SD* = 1.66 year). We recruited healthy adults with normal or corrected‐to‐normal vision through advertising on student Facebook pages and college noticeboards. They gave informed consent and were compensated for their time (rate of £10 per hour).

#### Children

The child sample consisted of 80 children (41 male, 39 female) with mean age 5.58 year (mean age in months was 67; *SD* = 4.03 month). After a primary state school agreed to participate, information letters with opt‐out forms were sent to parents of children in their first year of primary education (“Year 1”). Children were included unless parents submitted opt‐out forms. The school is located in an area ranked within the lowest country decile against an index of multiple deprivation factors (i.e., least deprived) and in the third‐lowest decile for income deprivation factors affecting children (Noble et al., 2019). The population in the school's area identifies itself as 84% white, 11% Asian, 3% black, and 2% mixed. The school is rated as “Outstanding” by the national authority inspecting schools.

#### Sample size

The Adults sample size was chosen based on a simulation‐based power analysis on previous data we collected with a comparable age range (Boettcher et al., 2021). We employed a conservative correction of the resulting effect sizes, and report power using a smallest effect size of interest that was 50% smaller than the actual effect sizes in our data. The analysis run with mixedpower (Kumle et al., 2018) showed that >24 participants would lead to a power of >80% even if our random sampling overestimated the actual population effects by 50%. The Children sample size was not predetermined, allowing everyone who wanted to be included (in line with our agreement with the school and the Ethics committee).

### Cognitive task: dynamic visual search

#### Apparatus

Children were tested in a dedicated room at their school. Young adults were tested in a testing booth at the Department of Experimental Psychology, University of Oxford. In both cases, participants sat in a quiet, dimly‐lit room, near a desk with the tablet on it. The tablet, a 10″ Microsoft Surface tablet (28 × 17 cm; 60 Hz refresh rate) running Linux, was placed within comfortable reach of the participant's hand. The tablet was standing at an upright position at 80° to the table by folding its cover. The experimental script was generated using Psychophysics Toolbox (Brainard, 1997) in MATLAB (version 2014b, The Mathworks Inc.).

#### Stimuli

**FIGURE 1:**
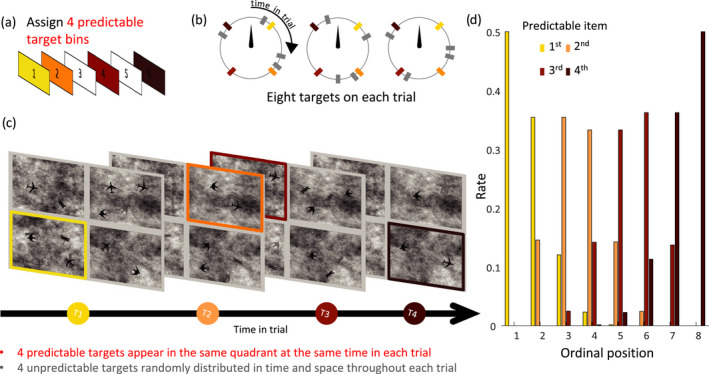
The sequence of events within a trial. (a) Each trial was divided into six time‐bins. Four predictable targets appeared in each trial, pre‐assigned in four of the six bins. (b) The colored dots represent predictable targets, which always occurred at the same time‐point within their assigned bin across a block of trials. The gray dots represent the four random targets, which were randomly distributed over time. (c) The four predictable targets were assigned to four different quadrants, which were kept constant throughout a block of trials. The quadrants in which random targets appeared were completely randomly determined. (d) The ordinal position of the predictable items within the sequence of eight targets. The histogram shows that items did not consistently appear in the same ordinal position, and in some cases (second and third predictable items) spread across five different ordinal positions

Figure 1 shows the task. Participants were instructed to find eight targets on each trial. Trials lasted approximately 14 s, and consisted of eight targets and sixteen distracting stimuli fading in and out of the search display independently over its duration. The search display consisted of four unique 1/F static noise patches that were generated for each trial. The four quadrants of the display were clearly separated, and each extended approximately 11° (horizontal angle) × 7° (vertical angle) (98 mm × 61 mm). Three different object stimuli were used as targets and distractors: an airplane, a dragonfly, and a bird. All stimuli were black with transparent backgrounds.

At the beginning of the experiment, each participant was randomly assigned one stimulus identity as a target (e.g., an airplane) and two stimulus identities from different categories as distractors (e.g., dragonfly and bird). Each of these three stimuli appeared eight times over the duration of each trial. In total, therefore, there were eight targets and sixteen distractors in each trial. Stimuli faded slowly in and out of view, but did not move. Fade‐in time was set to 1.3 s (gradually becoming visible over 80 refresh‐rate cycles until reaching maximum visibility). Each stimulus stayed on the screen for another 1.3 s, and then faded out over 1.3 s. Each stimulus was about 2 cm in length and width (roughly 2.5° at a mean distance of 45 cm) and could appear anywhere in one of the four quadrants as long as it did not overlap with another stimulus. On a given trial, each stimulus was assigned a unique location, that is, items could not appear twice at the same location. Participants tapped on the location of the target using the touch screen to indicate when they detected a target. Tapping a stimulus did not change its course of appearance and there was no immediate feedback. We instructed participants to tap on the targets, and there was no instruction regarding the total number of times an individual could tap on a target or within a trial.

#### Target predictability

The dynamic visual‐search task manipulated the spatiotemporal predictability of target stimuli. Of the eight target stimuli, four were predictable. On every trial, they appeared with the same temporal onset from the start of the trial and within the same quadrant. Location within the quadrant, however, remained variable. The onset times for predictable targets were pre‐assigned as follows: we created six time windows of ~1 s, with a gap of ~660 ms between each neighboring window (440–1440, 2100–3110, 3800–4800, 5470–6480, 7140–8140, 8810–9810 ms; times are relative to the onset of the trial, and rounded to the nearest 10 ms). Each window was then sub‐divided into four equally spaced onsets. At the beginning of the experiment, each participant was allocated four different predictable onsets by first picking four bins out of six possible ones; and then choosing one onset (out of four) within each bin. Once these intervals and their quadrant association were selected, they remained fixed for that participant throughout the experiment. Different participants had different interval‐quadrant pairings for predictable targets. The four unpredictable targets were distributed pseudorandomly, by assigning each to an onset derived from a uniform distribution across the full trial length. These constraints were set such that the targets were roughly evenly distributed throughout the trial, and to avoid too many target events occurring at one time.

Importantly, spatiotemporal predictability of targets could not be explained merely by sequential (or temporal order) effects or by differences in spatial attention alone. The use of random targets that appeared at any time and place prevented the sequential predictability of predictable targets. The ordinal position of predictable targets differed over trials. For example, the second predictable target could appear either as the 2^nd^/3^rd^/4^th^/5^th^ target in the sequence (thereby reducing contributions of sequential order), and the intervening items could happen anywhere (thereby reducing contributions of spatial attention). The highly variable overall distribution of target locations and timings between trials made it impossible to learn about regularities by simply learning about sequences or spatial locations. It is also worth noting that the pure contribution of spatial attention was diminished by spatial predictability being restricted to the level of a quadrant.

In Figure 1d, we plot a histogram with the ordinal position of each predictable target. For example, as shown in the figure, the second predictable items were as likely to appear as the second or the third, and often appeared also as the forth item. None of the items appeared at the same position for more than 50% of the trials.

#### Distractor predictability

In order to test for sensitivity to spatiotemporal regularities in unattended stimuli, half of the distractors were also made predictable for one of the distractor categories (e.g., within the dragonfly but not bird category). Predictability for these distractors was assigned in the same way as for the target category, but independently (allowing some targets and some distractors to overlap in time, in some cases). Thus, there was no systematic relation between target and distractor predictability.

#### Experimental procedure

Participants completed 10 practice trials to familiarize themselves with the game before completing a single block of 45 trials. Each trial contained eight targets (4 predictable and 4 unpredictable). Overall, there were 360 targets (180 predictable and 180 unpredictable) per participant (excluding practice trials) and 720 distractors (180 predictable and 540 unpredictable). After each trial, observers received feedback in the form of a visual illustration: The screen depicted eight targets in a horizontal row that were colored black for each target found and light gray for each target missed. These eight figures rotated, and a cheering sound was played when all the targets in a trial had been found. The feedback remained on the screen until tapping. When testing adults, they tapped to proceed at their own pace. When testing children, the experimenter tapped when the child was ready. The experimental block lasted approximately 15 min.

#### SWAN questionnaire

Teachers completed the Strengths and Weaknesses of ADHD‐symptoms and Normal‐behaviors (SWAN) rating scale for each (Swanson et al., 2012). This is a well‐validated scale with 18 items measuring ADHD symptoms, with each item scored from −3 to +3 (below average to above average). Lower scores correspond to higher ADHD characteristics. The SWAN questionnaire is not optimized for clinical assessment but rather was designed to capture variation across the neurotypical to atypical continuum and thus does not have a cut‐off scores for exclusion (see Swanson et al., 2012, for a rationale). We used a distribution‐based cut‐off score to identify outliers. One individual had a total ADHD score that exceeded three standard deviations compared to the mean (*Z *= −3.22; Raw score −54), and their SWAN data were therefore excluded from the analysis of individual differences (see Analysis section below).

#### Analyses

##### Dependent variables

Our primary‐dependent variable of interest was hit rate. We focused on hit rate, rather than reaction times, to avoid placing pressure on children and to avoid possible differences in response‐time variability between the groups introduced by the use of tablets. In our previous study with young adults (Boettcher et al., 2021; Shalev, Boettcher, et al., 2019), we found that hit rate provided a sensitive and reliable marker of performance in dynamic visual search. Accordingly, we did not instruct our participants to respond quickly. In addition, to assess the effect of predictability on distractors, we also considered false alarm rates (i.e., tapping on distractors rather than targets). Finally, we also consider whether predictions affected *multiple taps*: that is, how often did participants tap on the same target more than once.

##### Group differences in performance—hit rate

Hit rate was evaluated as a function of group (children vs. adults) and target predictability (predictable vs. unpredictable) to learn whether regularities improved children's and/or adults’ performance, whether the groups differed in their overall performance, and whether there was an interaction between group and predictability. In addition, we modeled “target order” to estimate behavioral effects over time on trial. This way, we could verify whether predictions influenced performance differentially for the first, second, third, or forth predictable versus random targets.

Responses to target, classified as “hit” or “miss,” were fitted using a generalized linear mixed‐effects models (GLMMs) with a binomial distribution. Data were modeled using three fixed effects: Group (children vs. adults); Target Predictability (Predictable vs. Random targets); and Target Order (First/Second/Third/Forth position in the sequence, for each Predictable vs. Random targets). In the random‐effects structure, we included intercepts for each participant, as well as by‐participant slopes for the effects of Target Predictability, Target Order, and their interaction.

Our model used the default “logit” link function to map the relation between the mean response and the linear combination of the predictors. The continuous Target Order factor was centered around zero by subtracting the grand average from each value (Aiken & West, 1991; Enders & Tofighi, 2007; Park, 2008). The GLMM was fitted with the maximum likelihood criterion, and we report the *t*‐statistic for a hypothesis test contrasting each fixed‐effects coefficient to a null hypothesis that the coefficient is equal to zero.

##### Group differences in performance—reaction times

For completion, we repeated the same modeling approach as in Hit Rate—this time fitting the reaction‐time data using a Linear Mixed‐Effect Model (LMM). Although participants were not instructed to respond quickly, and we did not control for hand position while using the tablet, we carried this secondary analysis to make sure there were no pronounced speed‐accuracy trade‐offs.

##### Group differences in performance—false alarms and multiple taps

We applied a comparable analysis approach to false alarms, by quantifying the rate of tapping distractors. Here, we tagged each distractor as belonging to one of three categories, to distinguish distractors that appeared predictably (“Structured category—Predictable”), distractors that appeared randomly but were from the same category as the predictable distractors (“Structured category—Random”), and distractors that appeared randomly and belonged to a different category (“Unstructured category—Random”). We used GLMM, with the Distractor Category, the Time Bin at which they appeared, and Group (children vs. adults) as the fixed‐effects. In the random‐effects structure, we included intercepts for each participant, as well as by‐participant slopes for the effect of Distractor Category, Time Bin, and their interaction.

Finally, we added a complementary analysis to estimate the effect of predictions on multiple taps. We applied a comparable model using a GLMM with Predictability, Time Bin, and Group as predictors of cases of tapping multiple times on a given target.

##### Individual differences

We applied a series of analyses to identify whether our dynamic visual‐search task was sensitive to individual differences *within* the group of children. In these analyses, we focused on the hit‐rate variable, which can reveal an interesting dissociation between “predictions” and overall hit rate. Two continuous variables were used to mark individual differences: chronological age (in months) and the combined SWAN score. The use of the combined SWAN score, rather than specific components (i.e., either inattention or hyperactivity traits) was motivated by the high correlation we observed between the two in our dataset (*r* = .88; *p *< .001).

The combined SWAN score and Age were added to a GLMM as fixed‐effects covariates, alongside Target Predictability. The random‐effects structure included intercepts for each participant, as well as by‐participant slopes for Target Predictability and their interaction. As in the previous section, the model used the default “logit” link function. The continuous variables (Age and SWAN score) were centered around zero by subtracting the grand average from each value (Aiken & West, 1991; Enders & Tofighi, 2007; Park, 2008). The GLMM was fitted with the maximum likelihood criterion, and we report the *t*‐statistic for a hypothesis test contrasting each fixed‐effects coefficient to a null hypothesis that the coefficient is equal to zero.

#### Data exclusion

Two children did not complete the experiment and were excluded. Altogether, our final sample included 78 children and 25 adults.

## RESULTS

### Contrasting group performance

**FIGURE 2:**
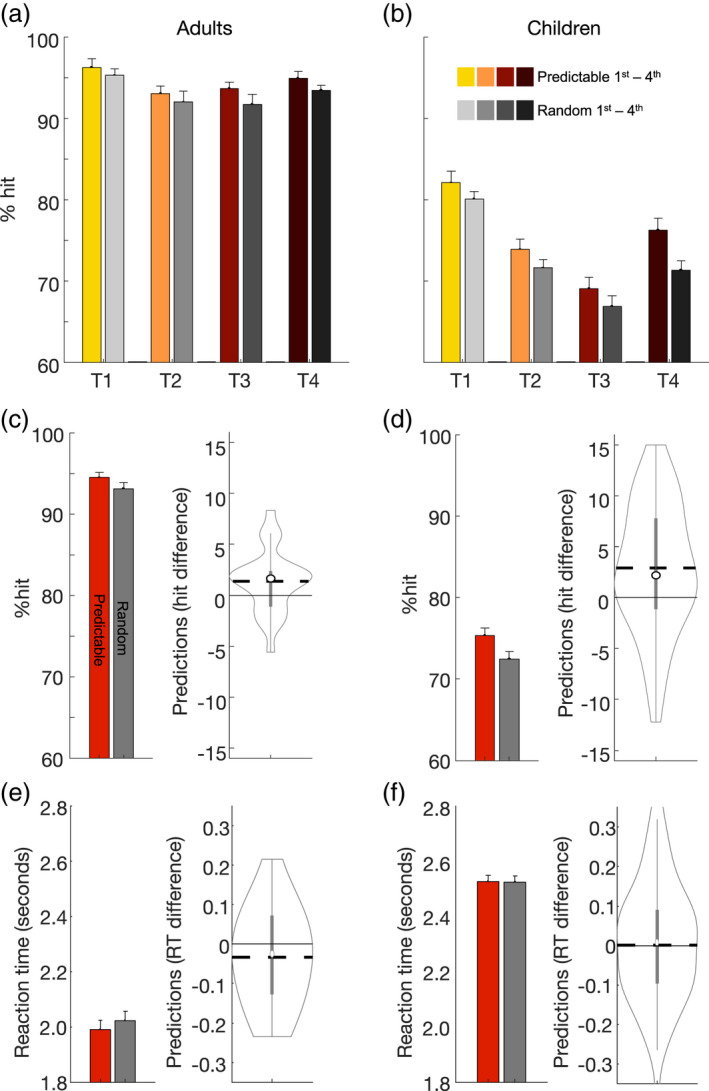
Performance on each group by conditions. (a) shows the hit rate of adults on predictable targets (colored bars) versus unpredictable (gray bars), splitting the trial into four equal time windows, to observe the benefit of predictions at different time‐points along each trial. Error bars, in all bar plots, represent standard error of the mean; (b) shows the same contrast among children; (c) overall hit‐rate for predictable (red) versus unpredictable targets (gray); the data in (d) depicts the difference between hitting predictable and unpredictable targets at the individual level, using a violin plot. The width represents the number of observations; The circle represents the group mean; horizontal dashed line represents the group median; bold vertical line is the interquartile range, and the thinner vertical line represents the range between higher and lower adjacent values; figures (e) and (f) depict the same as (c) and (d) among the group of children

Our first analysis focused on the contribution of target predictability and serial position to hit rate, according to group. The results are illustrated in Figure 2. The GLMM indicated a main effect of Target Predictability (*t* (37,072) = 3.99; *p *< .001; 95% CI [.05; .16]), a main effect of Group (*t* (37,072) = 16.33; *p *< .001; 95% CI [.75; .96]), and a main effect of Target Order (*t* (37,072) = −4.80; *p *< .001; 95% CI [−.16; −.06]). There were no interactions between factors. Group did not interact with Target Predictability (*p *= .38) or with Target Order (*p *= .23). Target Order did not interact with Target Predictability (*p *= .65), and there was no three‐way interaction among the factors (*p *= .87). Altogether, these results reflect a higher hit rate when targets appeared predictably, a higher hit rate in the group of adults, and differences in the mean hit rate at different time points along the trial. Critically, there were no interactions, meaning there were no evidence that either the group or the ordinal position of the target influenced performance benefits.

For completeness, we repeated a similar modeling procedure, this time with Reaction Times as the dependent variable. The results are illustrated in Figure 2. The LMM indicated a main effect of Target Predictability (*t* (29,152) = 2.18; *p *= .029; 95% CI [.00; .07]), a main effect of Group (*t* (29,152) = 13.556; *p *< .001; 95% CI [.41; .55]), and a main effect of Target Order (*t* (29,152) = 4.01; *p *< .001; 95% CI [.03; .09]). There were no interactions between the factors. Group did not interact with Target Predictability (*p *= .082) or with Target Order (*p *= .055). Target Order did not interact with Target Predictability (*p *= .75), and there was no three‐way interaction among the factors (*p *= .10). Altogether, these results reflect faster reaction times when targets appeared predictably, faster reaction times in the group of adults, and differences in the mean reaction times at different time points along the trial. There were no significant interactions, meaning there was no evidence that either the group or the ordinal position of the target influenced performance benefits.

**FIGURE 3:**
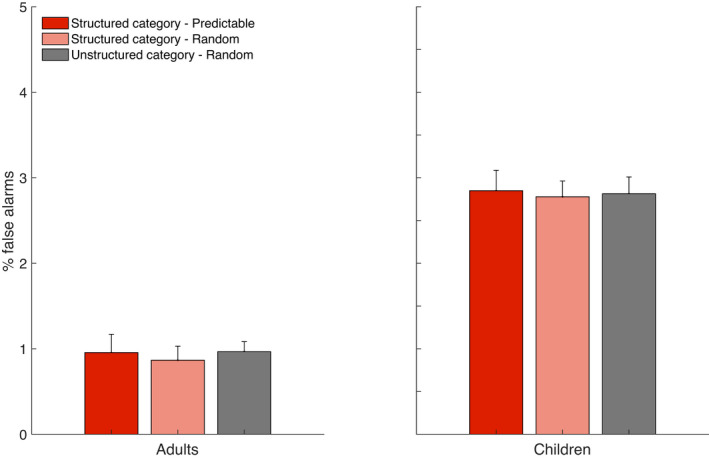
False‐alarm errors (selecting distractors instead of targets), split by three experimental conditions in two groups. Distractors that belonged to the “Structured category—Predictable” (red) are four distractors that appeared regularly, similar to the predictable targets; The “Structured category—Random” (light coral) were four distractors of the same category as the predictable ones (either airplanes, birds, or bugs), however, they were randomly distributed in time and space; the “Unstructured category—Random” were the other eight targets, of a different category, and were all distributed randomly. Error bars represent the standard error of the mean

The next analysis used comparable modeling to contrast the rate of false alarms in the two groups by Distractor Category and Time Bin. The GLMM results for the rate of false alarms indicated a main effect of Group (*t* (74,148) = 8.64; *p *< .001; 95% CI [.83; 1.32]), reflecting a significantly higher rate of false alarms for children. There was no main effect of Distractor Type (*p *= .85), no effect of Time Bin (*p *= .28), nor any significant interaction (all *p*s > .075). The main effect of group, and the rate of false alarms on each category, are illustrated in Figure 3.

Finally, we analyzed the proportion of multiple taps: cases where participants tapped on a target multiple times. Among young adults, 7.3% of responses were multi‐taps (*SE* = .002), and among children this percentage was 6.6% (*SE* = .001). We modeled the proportion of multi‐taps using a Generalized Linear Mixed‐Model, with Group and Target Predictability as independent variables, and found no significant effects (all *p*s > .32; with Group factor at *p *= .88).

### Individual differences among children

**FIGURE 4:**
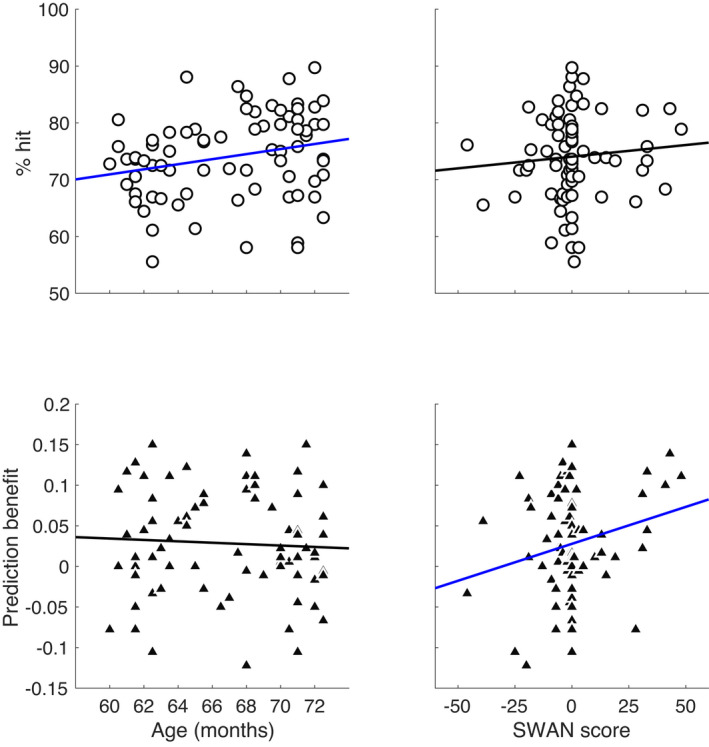
Scatter plots testing the association between performance measures, age, and SWAN score. Top row shows relations involving the overall mean hit rate. Overall hit rates correlated significantly with age but not SWAN score. Bottom row shows relations involving prediction benefit. Prediction benefit correlated/covaried significantly with SWAN score (.047) but not age (.25)

We tested for individual differences in overall hit rate and benefits of predictions in the group of children. The data are presented in Figure 4a–d, in which scatter plots depict the relations between the SWAN and Age factors as predictors of overall hit rate and of “prediction benefit” (defined as the difference between hit rate for predictable and unpredictable targets). Descriptive statistics of the SWAN questionnaire appear in Table 1.

**TABLE 1:** Descriptive statistics, summarizing the SWAN questionnaire data (total score and ADHD factors: inattention and hyperactivity/impulsivity)

Factor	Mean	*SD*	Min	Max
Total score	.48	15.8	−46	48
Inattention	−.33	7.6	−23	24
Hyperactivity/impulsivity	.81	8.6	−23	27

The statistical analyses tested for significant linear relations between Target Predictability (Predictable vs. Random), age (in months), teachers’ scores on the SWAN questionnaire, and the hit rate as the dependent variable, using a single GLMM. The results replicated the main effect of Target Predictability when focusing exclusively on the group of children (*t* (27,712) = 4.09; *p *< .001; 95% CI [.04; .11]). There was also a main effect of the Age (in months) covariate (*t* (27,712) = 2.26; *p *= .02; 95% CI [.00; .04]), with a positive coefficient (.025; *SE* = .01) indicating a positive relation between advancing age and overall hit rate. Whereas there was no main effect of SWAN score (*p *= .56), there was a significant interaction between the SWAN and Target Predictability (*t* (27,712) = 2.59; *p *= .009; 95% CI [.000; .005]). Age did not interact with Target Predictability (*p *= .75), and there were no other significant interactions (all *p*s > .1). To interpret the interaction between SWAN and Target Predictability, we calculated a composite score for Prediction Benefit by subtracting the hit rate for random targets from predictable ones. We then carried a confirmatory analysis to estimate the linear correlation between the two variables, which was positive and significant (*r* (77) = .23; *p *= .047), indicating a greater benefit of predictability for individuals with higher SWAN score (lower ADHD symptoms). Importantly, Age and SWAN score did not correlate (*r* (77) = .13; *p *= .25).

## DISCUSSION

Although young children showed the lower overall ability of detecting targets and ignoring distractors compared to adults in our extended dynamic visual‐search task, they nevertheless benefited from spatiotemporal regularities as much as adults. Our results suggest a more nuanced interpretation of how spatial guidance of attention operates among young children: while traditional studies highlight the immaturity of goal‐directed attention, we found that learning and experience influence attentional guidance strongly, even at this young age. In the context of our task, benefits from spatiotemporal regularities were selective in benefitting performance toward goal‐relevant target stimuli, and to an equivalent extent in children and adults. Children, like adults, did not show any bias toward task‐irrelevant regularities, while at the same time they learned relevant regularities that served anticipation‐based guidance.

Differences in overall visual‐search performance were reflected in the overall lower hit rate and higher false‐alarm rate in children when compared to adults. We also identified a significant correlation between hit rate and age within the group of children. This provides further and finer‐grained evidence of how visual search develops during childhood. This pattern may suggest particular difficulty with competition and distraction among children, in line with existing literature (Kim & Kastner, 2019). We observed that the lowest performance levels occurred in the middle of trial (reflected in the main effect of Target Order) when, overall, the likelihood of distractors peaked. An interesting possibility, therefore, is that decrements in performance are caused by maximum competition among stimuli around the middle of the trial due to the dynamic nature of the task. It was not possible to test the impact of distractor competition directly in our experiment, since multiple dynamic factors were at play which were not systematically controlled (location, number, and opacity of distractors and targets on the screen at any given moment). However, it is of note that target order did not significantly interact with predictability and even during periods of low performance both children and adults exhibited a benefit for predictable compared to unpredictable targets. This provides some indication that the predictability effect did not depend on the amount of distraction. Even so, addressing the influences of various aspects of competition on performance in dynamic search should be pursued in future tasks.

In contrast to age differences in overall hit rate in visual search, children showed reliable and persistent benefits of spatiotemporal expectation, which did not differ in magnitude from those we found in young adults. This pattern extends previous reports on the ability of children to learn patterns and utilize predictions. Previous work focused on discrete stimulus‐interval associations in cueing tasks as a source of anticipation (e.g., Droit‐Volet & Coull, 2016; Johnson et al., 2015; Mento & Granziol, 2020; Vallesi & Shallice, 2007). In the statistical learning literature, learning of visual regularities is normally confined to single objects and shape co‐occurrence, while temporal intervals are kept constant (e.g., Fiser & Aslin, 2002; Kirkham et al., 2002; Wu et al., 2011). Here we demonstrate that children can learn patterns of task‐relevant spatiotemporal information presented among dynamic distractors, which in turn can guide anticipation and performance benefits. We measured the spatiotemporal memories implicitly through the predictability benefits conferred to behavior. We did not assess whether children or adults became aware of the spatiotemporal structures embedded in the task. Although it is possible for unconscious memories to guide behavior, we cannot rule out at least some participants having partial or full awareness of the spatiotemporal structures. The nature of the memories guiding task benefits should prove a particularly interesting point to follow up from a developmental perspective. Previous developmental work has shown a dissociation between explicit and implicit use of timing cues (Droit‐Volet & Coull, 2016). In that study, while children and adults were comparable in their capacity to use time implicitly, they differed in their ability to make explicit reports of timing parameters (Droit‐Volet & Coull, 2016). Future work could investigate awareness of dynamic spatiotemporal regularities across development.

Another important addition we make to the current literature on anticipation‐led behavior in children pertains to the nature of structures that are learned and utilized. In our dynamic task, the behavioral benefit resulted from encoding temporal *intervals* (in contrast to serial order, as often used in studies of statistical learning). Furthermore, the regular temporal intervals were embedded within, and had to be extracted from, the context of many intervening unpredictable stimuli occurring across time and space. Learning single simple temporal associations would therefore be insufficient to confer any behavioral advantage. Instead, to benefit performance, participants had to abstract the timing and location of predictable relevant events from the unfolding succession of unpredictable targets and distractors. In addition, orienting of attention combined the temporal and spatial dimensions, in contrast to most previous studies on temporal attention in which target location is equated across trials—that is, the discrete events occurring at a consistent location.

Interestingly, even though children were overall worse than adults, task irrelevant, yet predictable stimuli did not yield more erroneous responses compared to their unpredictable counterparts. This is in contrast to previous reports of automatic capture by regularities (Zhao et al., 2013). In our task, such an effect would have been manifested by a larger false‐alarm rate for predictable distractors. If children were particularly susceptible to predictions, their capture by predictable distractors might be even stronger. This was not observed. We cannot completely rule out the possibility that our pattern of predictability was either too subtle or too complex to induce capture by task‐irrelevant information. However, overall our findings accord with studies in adults showing that effects of predictions are strongly modulated by task goals (Boettcher et al., 2020; Shalev, Nobre, et al., 2019; Stokes et al., 2014). What is somewhat surprising is that we also observed these same patterns in young children. In our task, instead of sensitivity to patterns working against focused, goal‐oriented behavior (Gaspelin et al., 2015), expectations only benefitted performance.

The benefit of expectations was tested within the group of children, in whom we modeled the data with two potential covariates: age and SWAN questionnaire (an ADHD symptom scale, used as a proxy of typical age‐appropriate attention development). The model results revealed a dissociation between task markers related to age (overall hit rate) versus with typical attention development (prediction benefits). Our findings suggested that the ability to form predictions does not change much with age (at least within the range between 5 and 6 years of age), but instead follows a proxy for age‐appropriate manifestation of attention. In the SWAN questionnaire, a lower score indicates a relative weakness on the ADHD scale. The negative interaction we found between the prediction factor and the SWAN score in our model, which was further verified using a simple Pearson correlation, indicates that children with relative weakness on the SWAN score were also less likely to rely on predictions when guiding behavior. While in the current study we did not target behavioral syndromes as ADHD, the data are in line with recent work showing difficulties in temporal preparation for adults diagnosed with ADHD (e.g., Dankner et al., 2017; Hasler et al., 2016; Johnson et al., 2008; McAvinue et al., 2015; Vallesi et al., 2016). However, direct implications of our data for an understanding of ADHD should be treated with caution. Our sample included relatively young children who had no reports of any clinical diagnosis, and therefore this point can be addressed further in future studies with more diverse and older samples.

In conclusion, we tested for the first time how children guide attention in a dynamic, continuous context, based on complex task‐embedded spatiotemporal regularities amidst distraction. In line with previous studies, children were less efficient in finding targets when compared to adults, but they used predictability to guide attention very effectively. This effect was robust and consistent throughout extended trials, revealing a marked capacity of encoding complex spatiotemporal structures, and sustaining attentional guidance to target dimensions over space and time. Moreover, we did not find capture by regularities embedded in task‐irrelevant information (predictable distractors), suggesting the ability of prediction‐based guidance based on trial history to support goal‐based attention in a selective manner. These robust effects were observed using a short, playful task, which can be a useful method for future explorations. One fruitful avenue could be investigating individual differences, which we managed to reveal within a single cohort of neurotypically developing 5‐ to 6‐year‐olds. We believe that our design provides a step forward toward understanding selective attention in ecological settings, in which the temporal dimension is bound to stimulus locations and identities.
